# Development of Functional Composite Edible Films or Coatings for Fruits Preservation with Addition of Pomace Oil-Based Nanoemulsion for Enhanced Barrier Properties and Caffeine for Enhanced Antioxidant Activity

**DOI:** 10.3390/molecules29163754

**Published:** 2024-08-08

**Authors:** Angelos-Panagiotis Bizymis, Virginia Giannou, Constantina Tzia

**Affiliations:** Laboratory of Food Chemistry and Technology, School of Chemical Engineering, National Technical University of Athens, 5 Iroon Polytechniou St., Polytechnioupoli, Zografou, 15780 Athens, Greece; apbizymis@yahoo.gr (A.-P.B.); vgiannou@chemeng.ntua.gr (V.G.)

**Keywords:** functional composite edible films, pomace oil-based nanoemulsions, caffeine, barrier properties, antioxidant activity, food packaging, strawberries, avocado

## Abstract

The aim of this study was to develop functional composite edible films or coatings for fruit preservation by the addition of bioactive components in combinations that have not yet been thoroughly studied, according to the relevant literature. Edible films were initially composed of (i) chitosan (CH), cellulose nanocrystals (CNC) and beta-cyclodextrin (CD) (50%-37.5%-12.5% ratio), and (ii) hydroxypropyl methylcellulose (HPMC), cellulose nanocrystals (CNC) and beta-cyclodextrin (CD) (50%-37.5%-12.5% ratio). The bioactive components incorporated (5, 10 and 15% *v*/*v*) were as follows: (i) pomace oil-based nanoemulsion (NE) aiming to enhance barrier properties, and (ii) caffeine (C), aiming to enhance the antioxidant activity of films, respectively. Indeed, NE addition led to very high barrier properties (low oxygen and water vapor permeability), increased flexibility and reduced color. Furthermore, the contribution of these coatings to fresh strawberries’ preservation under cold storage was investigated, with very promising results concerning weight loss, color difference, and preservation of fruit moisture and quantity of O_2_ and CO_2_ inside the packages. Additionally, C addition led to very high antioxidant activity, reduced color and improved barrier properties. Finally, the contribution of these coatings to avocado’s preservation under cold storage was investigated, with very encouraging results for color difference, hardness and peroxide value of the fruit samples.

## 1. Introduction

Food safety is a critical issue for the modern industry. In recent decades, consumer concerns about food processing and packaging methods and their effects on health, safety and the environment have increased. Consumers are looking for healthy, safe and highly nutritious foods, with extended shelf life and the least possible processing. Their requirements become even stricter concerning perishable foods such as meat, fish, fruits and vegetables. Thus, in order to meet growing demands, food technology is directed towards new preservation and packaging techniques [1,2,3].

The use of edible packaging is among the most prevalent modern tools developed in this direction. In fact, the market share of edible films and coatings is constantly increasing, as is the research in this field, which aims to improve their properties and enhance food preservation [3,4,5].

Plain edible films and coatings, made from a single basic material, meet certain requirements, such as barrier properties, attractive appearance of the products, increase in their shelf life, etc. However, efforts are being made to develop innovative and more advanced techniques, combining multiple materials (composite films/coatings). Particularly, in the case of active composite edible films and coatings, their functionality relies on the interaction between their components, the coated food product and the environment [6,7,8].

Two main classes of ingredients that can be incorporated into edible films and coatings to improve their characteristics are nanoemulsions and antioxidants.

Due to the increased lipid concentration, functional edible films and coatings containing nanoemulsions exhibit satisfactory mechanical properties (mainly in terms of flexibility), high transparency and greater homogeneity [9,10]. Furthermore, the addition of nanoemulsions results in the decrease in both water vapor permeability and oxygen permeability at high relative humidities (>0.75%), despite a slight increase in the latter at low relative humidities [11,12,13,14,15,16,17]. Nanoemulsions also have the ability to encapsulate various functional compounds and active ingredients [18,19]. Edible nano-films/coatings containing flavor and colorants, antioxidants, enzymes, antimicrobials and enzyme browning inhibitors can be applied on various food products (e.g., meat, dairy products, fruits, vegetables and pastry products) [20,21,22].

The oxidation of food lipids can cause off odors, undesirable texture and color changes (e.g., discoloration due to myoglobin oxidation). In addition to these sensorial changes, toxic compounds, such as aldehydes, can also be formed, loss of vitamins and nutrients can occur, and ultimately, the shelf life of the products can be reduced [23,24,25]. For these reasons, the main purpose of incorporating antioxidants into edible films and coatings is to delay lipid oxidation. This method is more effective than the direct addition of antioxidants to the food since they are applied on its sensitive surface together with the film or coating, and it is also more economical since it requires significantly smaller amounts of antioxidants [26,27]. In fact, as consumers turn more and more to natural products, the use of natural antioxidants is preferred over traditional synthetic ones [23,28,29]. Indeed, the selection of the appropriate active agent is a critical step in the preparation of a functional film or coating [13,27,30]. The antioxidant compounds and the coating materials should be compatible with each other in order to achieve a homogeneous distribution of the antioxidants and consequently the most effective protection of the entire surface of the food. In the last decade, many edible materials containing antioxidants have been tested in food products, such as fresh fruits or vegetables, cheese, meat and fish [23,28,29,31,32].

One material that can provide antioxidant activity to edible films and coatings is caffeine (C). C is a xanthine alkaloid, found in various parts of plants, such as coffee and cocoa beans, tea leaves, guarana fruits, etc. In plants, it acts as a natural pesticide, protecting them from insects that feed on them, while in humans, it acts as a stimulant of the central nervous system by increasing dopamine levels [33,34]. C is widely used and consumed as a food and beverage additive (e.g., in coffee, tea, etc.) [35,36]. It is the most widely used psychoactive substance in the world, but, unlike many other psychoactive substances, its use is legal and free in almost all countries. Furthermore, when used as an ingredient, C is not classified as an addictive substance [37]. The Food and Drug Administration (FDA) describes C as a “multipurpose generally recognized as safe food substance” [38]. The safe intake of C is defined by the European Food Safety Authority (EFSA) at 400 mg/day. Thus, in addition to its antioxidant effect, the reasonable use of caffeine can offer several benefits to the human body, since it can improve mood, reflexes, memory and alertness [33,34,39].

The present study examines the incorporation of pomace oil-based nanoemulsion (NE) and of C into two types of innovative composite edible films/coatings, which have been introduced in previous studies of Bizymis et al. [8,40,41] and have shown remarkably improved properties (e.g., high oxygen and water vapor barrier and high mechanical structure). The initial solutions consisted of the following: (i) chitosan (CH), cellulose nanocrystals (CNC) and beta-cyclodextrin (CD), and (ii) hydroxypropyl methylcellulose (HPMC), cellulose nanocrystals (CNC) and beta-cyclodextrin (CD). More specifically, the previous studies of Bizymis et al. [8,40,41] showed that these innovative (according to the relevant literature) ternary combinations created films with enhanced properties, which appeared to have great prospects for wider application. In particular, in the CH-CNC-CD case, the addition of both CNC and CD to the CH base material, in all ratios, improved most of the properties examined. Increasing the CD content resulted in better optical properties (lighter color and increased transparency), while increasing the CNC content resulted in better coating characteristics (increased surface tension and reduced viscosity) and superior mechanical properties (elastic modulus and breaking stress). Also, both additions remarkably improved the barrier properties of the formed films. Likewise, the addition of both CNC and CD to the HPMC base material contributed to the overall improvement in the properties of the final films. CD further improved the barrier and optical properties, while CNC led to better coating characteristics and to higher mechanical properties. Apart from these, in the previous studies of Bizymis et al. [40,41], scanning electron microscopy (SEM) images and Fourier-transform infrared (FTIR) spectra of the surface of the various films were presented, which, along with the rest of the results, led to the selection of the 50%-37.5%-12.5% ratio for both CH-CNC-CD and HPMC-CNC-CD solutions for the purpose of the current study.

Following the above, in the current study, (i) NE was incorporated in alternative proportions in the CH-CNC-CD and HPMC-CNC-CD solutions (50%-37.5%-12.5% ratios in both cases), in order to examine the barrier properties of the final functional edible films/coatings and the effect of their application in strawberries preservation, and (ii) C was incorporated in alternative proportions in the CH-CNC-CD and HPMC-CNC-CD solutions (at the same ratios), with the objective of examining the antioxidant activity of the final functional edible films/coatings and the effects of their application in avocado preservation.

It must be noted at this point that during the study of the relevant literature, different combinations and alternative applications of the aforementioned components were identified, which supports the claim about the innovative character of the films prepared in the context of the current research. Some indicative examples from the literature are as follows:

- Improved barrier properties by the addition of gelatin to CH films and application of the films on red peppers to extend their shelf life [42].
- Enhanced antioxidant activity by the incorporation of *Tricholoma terreum* extract in CH films [43].
- Improved mechanical stability and decreased oxygen permeability by the addition of nanoclay to HPMC films [44].
- Improved mechanical stability and decreased water vapor permeability by the addition of CNC to carboxymethylcellulose-based films [45].
- Low water-vapor permeability and high transparency of films combining HPMC with nisin, potassium sorbate and tapioca starch [46,47].

In conclusion, the purpose of the present study is to further extend the previous research of Bizymis et al. [8,40,41] on the innovative combinations of CH-CNC-CD and HPMC-CNC-CD in edible films and coatings. Τhe incorporation of NE or C in the above ternary combinations has not yet been thoroughly analyzed. Therefore, the purpose of the present study is to investigate these novel combinations in detail, to highlight their advantages, and to support further research for their commercial application.

Finally, unlike most studies that emphasize only on the coated food products [4,7,48,49,50,51,52,53,54,55,56,57,58], the present research also studies and examines the characteristics and properties of the composite edible films themselves prior to their application on fresh fruits (strawberries and avocado).

## 2. Results and Discussion

### 2.1. Properties of the Functional Composite Edible Films with Incorporation of NE

In this section, the incorporation of NE into CH-CNC-CD and HPMC-CNC-CD composite edible films (both having ratios of 50%-37.5%-12.5%) was investigated, with the main objective of improving their barrier properties.

#### 2.1.1. Mechanical and Optical Properties

For both film types, the following parameters were measured (Table 1):

- Maximum breaking force (F) and percent of elongation at break (ε) regarding the mechanical properties;
- Color difference from a white plate (Δ*E*) and chrome (C*) regarding the optical properties.

Regarding the mechanical properties, according to Table 1, before NE incorporation, both types of films were quite flexible and exhibited high mechanical strength, which is in agreement with similar studies [40,41,59,60,61,62,63,64]. However, the mechanical properties of CH-CNC-CD films were to some extent superior to those of HPMC-CNC-CD (F was 19.31 N in CH-CNC-CD films and 13.44 N in HPMC-CNC-CD films, and the ε index was 16.78% in CH-CNC-CD films and 14.08% in HPMC-CNC-CD films). 

In accordance with the above, experiments described in Bizymis et al. [40,41] showed that plain films with 1% *w*/*v* CH presented higher mechanical stability than those with 1% *w*/*v* HPMC (F was 23.94 N in the plain CH films and 14.29 N in the plain HPMC films), apparently because CH is a material of higher mechanical strength than HPMC. Furthermore, the results in Table 1 show that the addition of CNC and CD most probably caused lower interactions between the molecules of the composite films, as it slightly degraded their mechanical strength compared to the plain CH and HPMC films [8,40,41].

By incorporating NE, the mechanical properties of the films were significantly differentiated (Table 1). In particular, F decreased (indicatively, for 15% *v*/*v* incorporation to 7.12 N in CH-CNC-CD films and to 3.31 N in those of HPMC-CNC-CD), while the ε index increased (for 15% *v*/*v* incorporation to 30.03% in CH-CNC-CD films and to 25.95% in those of HPMC-CNC-CD). This indicates that NE made the films more flexible but at the same time less durable. The explanation for this lies in the different interactions between polymers and nanoemulsions. Τhe weaker interactions between polymers and NE resulted in the formation of discontinuities, especially at high NE concentrations, which created ruptures in the matrix of the films and therefore lower mechanical strength [7,22].

Regarding the optical properties (Table 1), the HPMC-CNC-CD films, without the incorporation of NE, showed a lower Δ*E* value than those of CH-CNC-CD (30.37 versus 47.59) and the same C* value (1.41). Overall, both types of films presented satisfactory enough optical properties and were quite transparent.

In accordance with the above, experiments described in Bizymis et al. [40,41] showed that plain films with 1% *w*/*v* CH were slightly superior in C* than those with 1% *w*/*v* HPMC (1.61 in plain CH films versus 2.23 in plain HPMC films) and slightly inferior in Δ*E* (31.99 in plain HPMC films versus 36.17 in plain CH films). Furthermore, the results in Table 1 indicate that due to the fine dispersion of CNC and CD in the CH and HPMC matrices, both types of composite films maintained their optical properties at satisfactory levels [65].

Upon the incorporation of NE, the C* values decreased (for 15% *v*/*v* incorporation, to 0.36 in CH-CNC-CD films and to 0.20 in those of HPMC-CNC-CD); thus, a lower color intensity was achieved, which is obviously a positive result [66]. On the other hand, Δ*E* improved in the case of CH-CNC-CD films (*p* < 0.05) (for 15% *v*/*v* incorporation, to 29.37) and slightly degraded in the case of HPMC-CNC-CD (for 15% *v*/*v* incorporation, to 35.10). Finally, NE incorporation made the films more transparent, because smaller particles disperse light to a lesser extent, thus reducing opacity [19].

#### 2.1.2. Barrier Properties

Table 2 presents the barrier properties and more specifically the oxygen permeability (*OP*) and water vapor permeability (*WVP*) of the film samples.

According to Table 2, both types of films, without NE, presented quite satisfactory values in *OP* and *WVP*, although CH and HPMC composite films tend to form crosslinks with water and to increase relative humidity [61,67,68,69]. In fact, HPMC-CNC-CD films were slightly superior to those of CH-CNC-CD in both properties (*p* < 0.05). 

Indeed, HPMC is a polysaccharide with a stable crystalline structure, which forms edible films with very good O_2_, CO_2_ and lipid barrier properties [8,30,41,61,62]. CH is a polysaccharide as well, making it biodegradable and compatible with other materials, and also forms edible films that are effective O_2_ and CO_2_ barriers [70,71,72]. 

In accordance with the above, experiments described in Bizymis et al. [40,41] showed that plain films with 1% *w*/*v* HPMC were superior to those with 1% *w*/*v* CH in both their *OP* and *WVP* properties (with values of 1.98·10^−12^ g·s^−1^·Pa^−1^·m^−1^ and 1.84·10^−9^ g·s^−1^·Pa^−1^·m^−1^, respectively, versus 2.99·10^−12^ g·s^−1^·Pa^−1^·m^−1^ and 2.20·10^−9^ g·s^−1^·Pa^−1^·m^−1^, respectively). Furthermore, the results in Table 2 indicate that the CNC and CD addition overall improved these properties. A possible explanation for their positive effect is that CNC nanomaterials, due to their high crystallinity and low hygroscopicity, create strong hydrogen bonds with other polymers, and especially with those that are hydrophilic and water-soluble [8,40,41]. Consequently, CNC increases the crystalline regions and in this way favors lower permeability for the edible films [73,74,75]. Additionally, due to its hydrophobic nature, CD also enhances the barrier against moisture and improves water resistance [60].

The incorporation of NE significantly decreased (*p* < 0.05) the *OP* and *WVP* values for both types of edible films. Indeed, this decrease was proportional to the amount of NE added (for 15% *v*/*v* incorporation in the CH-CNC-CD films, a reduction in *OP* and *WVP* by 46% was observed, while in those of HPMC-CNC-CD, the reduction in *OP* was by 35% and in *WVP* by 34%). In fact, the incorporation of NE increased the hydrophobic character of the films [7]. These results are attributed to the fact that NE creates greater homogeneity in the structure of the films, thus reducing the amorphous regions that favor the transport of water and oxygen [9,18,76].

It is also worth noting that compared to the initial plain 1% *w*/*v* CH films reported by Bizymis et al. [40,41], CH-CNC-CD films with NE incorporation showed an up to 66% reduction in *OP*, reaching 1.02 × 10^−12^ g·s^−1^·Pa^−1^·m^−1^, and up to 45% for *WVP*, reaching 1.22 × 10^−9^ g·s^−1^·Pa^−1^·m^−1^. Correspondingly, NE-incorporated HPMC-CNC-CD films showed an up to 47% reduction in *OP*, reaching 1.04 × 10^−12^ g·s^−1^·Pa^−1^·m^−1^, and up to 39% for *WVP*, reaching 1.13 × 10^−9^ g·s^−1^·Pa^−1^·m^−1^ compared to the initial plain 1% *w*/*v* HPMC films also reported by Bizymis et al. [40,41].

Additionally, as reported in the literature, an 8% addition of nanoclay in HPMC films decreased their *OP* by 21% [44], while the incorporation of nisin, potassium sorbate and tapioca starch, decreased their *WVP* values at 1.85 × 10^−9^ g·s^−1^·Pa^−1^·m^−1^ [46,47]. Similarly, adding 5% CNC to carboxymethylcellulose-based edible films resulted in an almost 54% decrease in their WVP values [45].

### 2.2. Properties of the Functional Composite Edible Films with Incorporation of C

In this section, the antioxidant capacity potential of CH-CNC-CD and HPMC-CNC-CD composite edible films (both in ratios of 50%-37.5%-12.5%) was investigated. For this purpose, the incorporation of C into the films was chosen, since its consumption, within limits, is considered beneficial for the human body. As already mentioned, the use of C in edible films has not been generally studied, according to the relevant literature.

#### 2.2.1. Mechanical and Optical Properties

The mechanical and optical properties examined in the film samples are presented in Table 3. 

The properties of the films that did not contain C have already been thoroughly discussed in Section 2.1.1.

Regarding the mechanical properties of the films, the incorporation of C did not significantly affect the CH-CNC-CD samples, while it improved, to some extent, those with HPMC-CNC-CD (Table 3). Indicatively, in the HPMC-CNC-CD films, the 15% *v*/*v* incorporation of C increased F to 18.67 N and the ε index to 16.28%. These findings are explained by the intermolecular bonds developed between C and the other components with an effect on the durability of the films [7].

As far as the optical properties are concerned, the C* values decreased (for 15% *v*/*v* incorporation, to 0.63 in CH-CNC-CD films and to 0.62 in those of HPMC-CNC-CD), due to C addition, resulting in lower color intensity. On the other hand, ΔE significantly improved in the case of CH-CNC-CD films (*p* < 0.05) (for 15% *v*/*v* incorporation, to 32.28) and remained at the same levels for HPMC-CNC-CD. This is due to the complete transparency of the pure C solution.

#### 2.2.2. Barrier Properties

Table 4 presents the oxygen permeability (*OP*) and water vapor permeability (*WVP*) of the film samples with and without C.

The barrier properties of the samples that did not contain C have already been thoroughly discussed in Section 2.1.2.

The incorporation of C significantly decreased (*p* < 0.05) the *OP* and *WVP* values for both types of films. In particular, the incorporation of 15% *v*/*v* C reduced *OP* by 37% and *WVP* by 39%, in the case of CH-CNC-CD films, while for HPMC-CNC-CD films the reduction in *OP* and *WVP* was 28% and 21%, respectively. An explanation for this is that C reduces the mean free volume and chain movement of the film-forming polymers and also changes the free volume distribution, thus reducing the areas of oxygen and water diffusion [33,77].

Furthermore, it should be noted that CH-CNC-CD films containing C showed an up to 60% reduction in *OP* (reaching 1.20 × 10^−12^ g·s^−1^·Pa^−1^·m^−1^), and an up to 37% reduction in *WVP* (reaching 1.38 × 10^−9^ g·s^−1^·Pa^−1^·m^−1^) compared to plain 1% *w*/*v* CH films reported by Bizymis et al. [40,41]. Finally, the incorporation of C in the HPMC-CNC-CD films reduced their *OP* up to 41% (reaching 1.16 × 10^−12^ g·s^−1^·Pa^−1^·m^−1^), and their *WVP* up to 26% (reaching 1.36 × 10^−9^ g·s^−1^·Pa^−1^·m^−1^) compared to plain 1% *w*/*v* HPMC films also reported by Bizymis et al. [40,41]. 

#### 2.2.3. Antioxidant Activity

Antioxidant activity is a very important factor against oxidative rancidity, spoilage and discoloration of food. Films and coatings with active antioxidant agents in their components prevent oxidation and contribute to extending the shelf life of food products. More specifically, antioxidants are stable molecules which can donate electrons to unstable molecules. In particular, they react with free radicals and reactive oxygen species (ROS), thus preventing the chain reaction that causes food spoilage. So, especially in the case of fresh-cut fruit, antioxidants act against enzymatic browning and increase consumers’ preference [78].

Table 5 presents the antioxidant activity values for the final edible films.

According to the results, the CH-CNC-CD films showed, in general, higher rates of antioxidant activity than those of HPMC-CNC-CD. The incorporation of C increased the antioxidant activity of both types of films (*AA* = 88.54% for 15% *v*/*v* incorporation of C in the CH-CNC-CD films and 81.19% in those of HPMC-CNC-CD), which is in accordance with the fact that C is an antioxidant substance. This is an important finding, since reasonable C intake can also provide positive effects to the consumer, such as increased stimulation and alertness, and improved mood, memory and reflexes [33,34,39].

As reported in the literature, the incorporation of *Tricholoma terreum* extract at 33% in CH films increased their antioxidant activity up to 84% [43], while in the present study with the incorporation of C in CH-CNC-CD films, it reached over 88%.

### 2.3. Investigation of the Application of Functional Composite Edible Coatings with NE on Strawberries

This section investigates the effectiveness of CH-CNC-CD and HPMC-CNC-CD coatings (both in ratios of 50%-37.5%-12.5%), with or without the incorporation of NE at 5% and 15% *v*/*v*, on strawberries. 

Strawberries are distinguished by their high content of biologically active components, such as phenolics (flavonoids, anthocyanins and ellagitannins), fibers, micronutrients (folic acid, vitamin C, lutein, zeaxanthin and choline), and minerals/inorganic elements (calcium, iron, magnesium, phosphorus, sodium, zinc, copper, magnesium and selenium). Because of these bioactive components, strawberries offer multiple health benefits. However, their pores favor the transfer of oxygen (O_2_) from the environment to their interior through the process of respiration and the transfer of water (H_2_O) from their interior to the environment, resulting in the rapid degradation of their quality. Thus, in the context of this research, the specific edible coatings were applied with the main purpose of reducing the permeability of both O_2_ and H_2_O. The calculation of the decrease in O_2_ permeability was based on the change in the amount of O_2_ and CO_2_ inside the package during the storage period, while the calculation of the decrease in H_2_O permeability was based on the change in the moisture of the samples during the storage period. Besides that, weight loss (*WL*), color difference (Δ*E*) and hardness were also studied at regular intervals.

#### 2.3.1. Weight Loss (*WL*) of the Strawberry Samples

**Figure 1:**
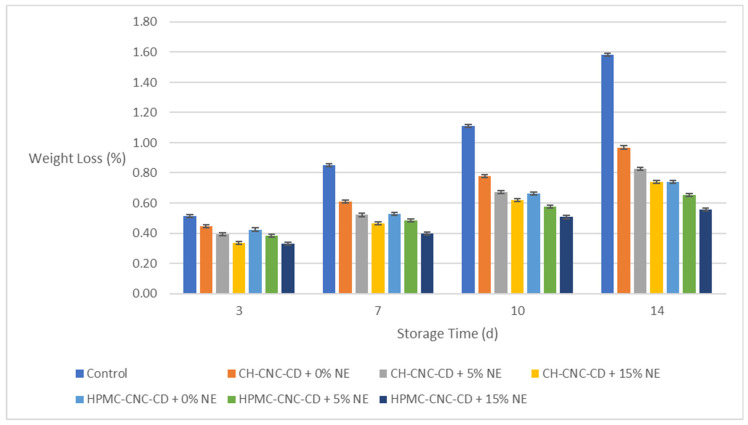
Change in weight loss (*WL*) of strawberries during storage.

As expected and reflected in Figure 1, the *WL* increased during storage. However, the difference in *WL* between uncoated (control) and coated samples is evident. In particular, samples with the NE-incorporated edible coatings showed lower *WL* in all cases, with the difference being noticeable as early as the 7th day of storage (in the NE-incorporated coated samples, *WL* generally remained below 0.80%, even on the 14th day, while in control samples, it reached 1.60% on the same day). This result is consistent with the *WVP* values measured in the films, as the main reason for the change in the weight of a food is the change in its water content. Also, samples with edible HPMC-CNC-CD coatings showed lower *WL* than those with CH-CNC-CD, which is consistent with the lower *WVP* values of HPMC-CNC-CD films (Section 2.1.2).

#### 2.3.2. Color Difference (Δ*E*) of the Strawberry Samples

**Figure 2:**
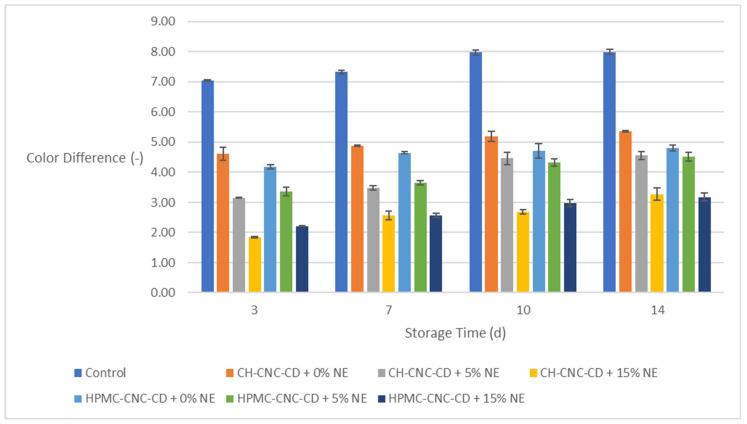
Change in color difference (Δ*E*) of strawberries during storage.

Color is an important parameter for the evaluation of food quality by consumers. For this reason, the color difference (Δ*E*) was evaluated for all types of strawberry samples, starting from the 3rd day of storage (Figure 2). The effect of the edible coatings in preserving the color of the samples is significant, since, in all phases, the control samples showed higher Δ*E* by at least three units compared to most coated ones. Moreover, the NE-coated samples, especially those with 15% *v*/*v*, showed even lower Δ*E* values (below five in all cases, even on the 14th day), which is explained by the reduced permeability of the surface, which allows less interactions with the environment and consequently smaller color changes and lower enzymatic browning. Finally, both CH and HPMC based coatings exhibited similar behavior.

#### 2.3.3. Hardness of the Strawberry Samples

**Figure 3:**
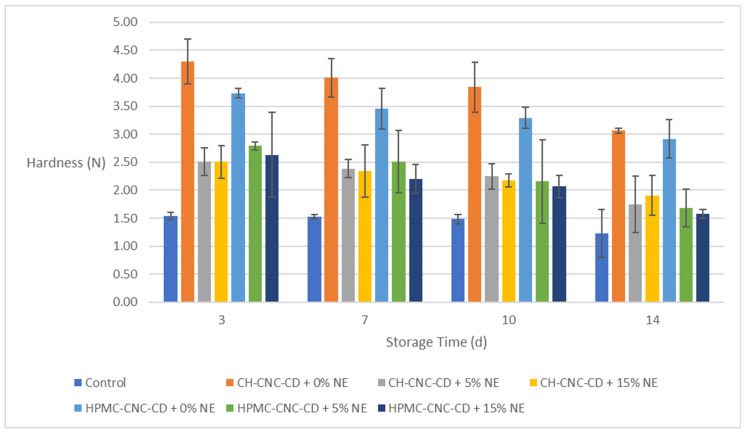
Change in hardness of strawberries during storage.

The average hardness of the control strawberry samples was measured at 2.022 N right before storage (day 0), while the evaluation of samples’ hardness started at the 3rd day of storage (Figure 3). It is evident that, in all cases, coated samples exhibited much higher hardness values compared to the control ones. This is obviously important for their shelf life, since increased surface hardness implies the maintenance of the integrity of the fruits and the preservation of their quality for a longer period of time. More specifically, upon their application, all coatings increased the hardness of the fruit surface. Certainly, over time fruit hardness decreased in all cases, but remaining higher for the coated samples. Moreover, samples coated with CH-CNC-CD without NE showed better results (above 3 N even on the 14th day) than the respective ones with HPMC-CNC-CD (throughout the storage period they were superior by approximately 0.20 N to 0.60 N), which is attributed to the higher mechanical strength of CH compared to HPMC. Finally, the NE-coated samples retained higher hardness than the control samples but were far behind those coated without NE. This result agrees with the previous finding that the addition of NE caused a decrease in the durability of the films (Section 2.1.1).

#### 2.3.4. Quantity of O_2_ and CO_2_ in the Packages of the Strawberry Samples

**Figure 4:**
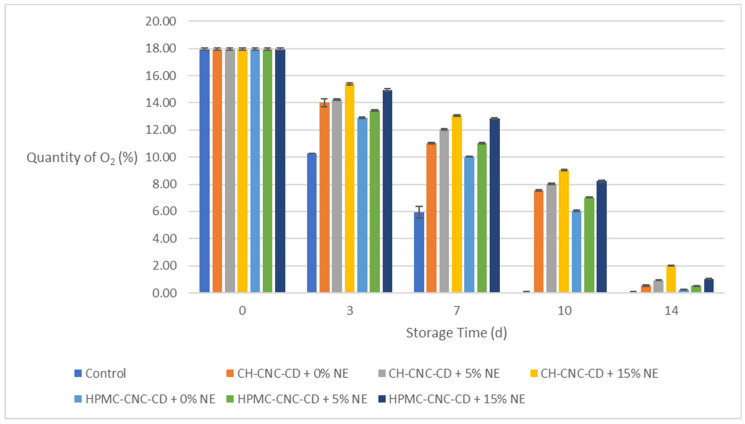
Change in quantity of O_2_ in the packages during storage.

**Figure 5:**
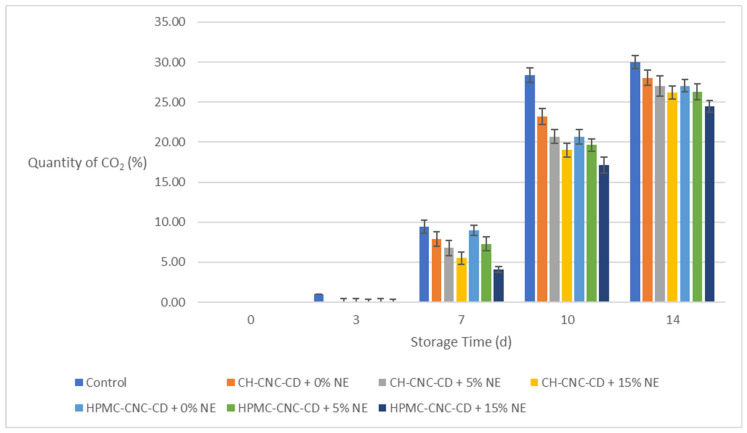
Change in quantity of CO_2_ in the packages during storage.

Figure 4 shows the changes in the amount of O_2_ in the packages during storage for all strawberry samples, and Figure 5 shows the changes in the amount of CO_2_, respectively. As shown in the diagrams, the quantity of O_2_ in the package decreases while the quantity of CO_2_ increases during storage due to the respiration process of the fruits. This is because O_2_ penetrates the food and is then expelled from it through CO_2_. The application of a coating greatly helps to limit this effect. In particular, samples with NE-containing coatings showed the smallest O_2_ decreases and, correspondingly, the smallest CO_2_ increases. Indeed, on the 10th day of storage, the amount of O_2_ was 0% and of CO_2_ more than 28% in the control samples, while on the same day, the respective amount of O_2_ was above 7% and of CO_2_ between 17 and 21% in coated samples with NE. This result is associated with the reduction in *OP* achieved by the coatings and additionally enhanced by the incorporation of NE (Section 2.1.2).

#### 2.3.5. Moisture (*M_w_*) of the Strawberry Samples

**Figure 6:**
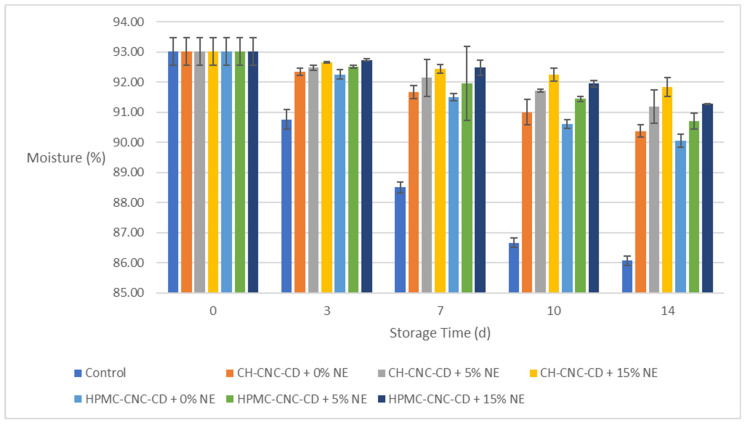
Change in moisture (*M_w_*) of strawberries during storage.

Figure 6 shows the changes in moisture (*M_w_*) during storage for all strawberry samples. As presented in the diagram, the *M_w_* of the fruits decreases during storage, apparently due to loss of H_2_O from their interior. The application of the coatings significantly contributed to the reduction in moisture loss. In particular, samples with coatings containing NE showed the smallest *M_w_* reductions in all cases, with the difference already noticeable on the 3rd day of storage. Indeed, even on the 14th day of storage, their M_w_ remained above 90%, while on the same day, the *M_w_* of the control samples had decreased to 86% from 93% on day 0. This effect is linked to the reduction in *WVP* achieved by the coatings, which is further enhanced due to the incorporation of NE (Section 2.1.2).

In conclusion, the edible coatings selected, and especially those containing NE, satisfied the basic purpose of reducing the permeability through the strawberries’ surface for both H_2_O and O_2_, thereby enhancing their preservation and quality.

#### 2.3.6. Statistical Analysis of the Results for the Strawberry Samples

Because of the involvement of several parameters (time, use of coating or not, type of base material, alternative NE incorporations), bar charts were chosen in the above sections to show more clearly the changes in samples’ properties over time.

Additionally, Table S1 in the Supplementary Materials presents the statistical analysis of the results based on storage time and the type of sample (control or coated). According to Table S1, the changes in all properties were statistically significant with respect to storage time and also proportional to it, except for Δ*E*, hardness and *M_w_* between the 10th and the 14th day of storage. However, it is worth noticing that the condition of all coated samples remained satisfactory until the end of storage.

As far as the type of sample (control or coated with each of the six different coatings) is concerned, it was obvious that the control samples were statistically significantly different from the coated ones in all properties examined. In most cases (*WL*, Δ*Ε*, quantity of O_2_ and CO_2_, *M_w_*), samples containing the highest NE proportion (15% *v*/*v*) exhibited significantly (*p* < 0.05) better values compared to the rest of the samples for both types of films (CH-CNC-CD and HPMC-CNC-CD).

### 2.4. Investigation of the Application of Functional Composite Edible Coatings with C on Avocado

This section investigates the effectiveness of CH-CNC-CD and HPMC-CNC-CD coatings (both in ratios of 50%-37.5%-12.5%) on avocado, with or without the incorporation of C at the levels of 5% and 15% *v*/*v*. Avocado is a tropical fruit that is widely produced and consumed. Its high nutritional composition and mild taste make it a unique and versatile ingredient in various culinary applications. However, it is strongly affected by the phenomenon of oxidation due to its high fat content (about 15–20%). Thus, in the context of this research, the main purpose of applying edible coatings was to provide samples with a satisfactory antioxidant activity. The calculation of the antioxidant activity of the coatings was based on the change in the number of peroxides in the avocado samples during the storage period. Besides that, weight loss (*WL*), color difference (Δ*E*) and hardness were also studied at regular intervals.

#### 2.4.1. Weight Loss (*WL*) of the Avocado Samples

**Figure 7:**
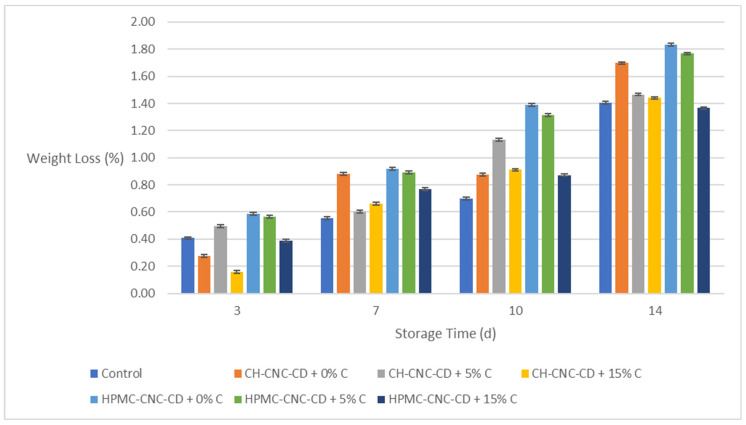
Change in weight loss (*WL*) of avocados during storage.

As shown in Figure 7, *WL* increases during storage. Samples with edible coatings without C showed unsatisfactory results (on the 7th day, the *WL* in the coated samples was more than 0.80%, while in the control samples, it was less than 0.60%). However, the addition of C improved the results, particularly at the end of the storage time. Specifically, on the 14th day, the *WL* of samples coated with C containing films was around 1.40%, as in the control samples, except for HPMC-CNC-CD coatings with 5% *v*/*v* C that did not show substantial improvement, while in the coated samples without C, it reached 1.70% and above. This result is consistent with the decrease in *WVP* values observed in the films after C incorporation (Section 2.2.2).

#### 2.4.2. Color Difference (Δ*E*) of the Avocado Samples

During storage, fresh fruits and vegetables show color changes and undergo enzymatic browning mainly due to the oxidation of their phenolic compounds. Indicatively, given the fact that oxygen is necessary for the polyphenol oxidase (PPO) to cause a browning reaction, edible food packaging with high oxygen barrier properties seems to be an effective tool against this process [79].

**Figure 8:**
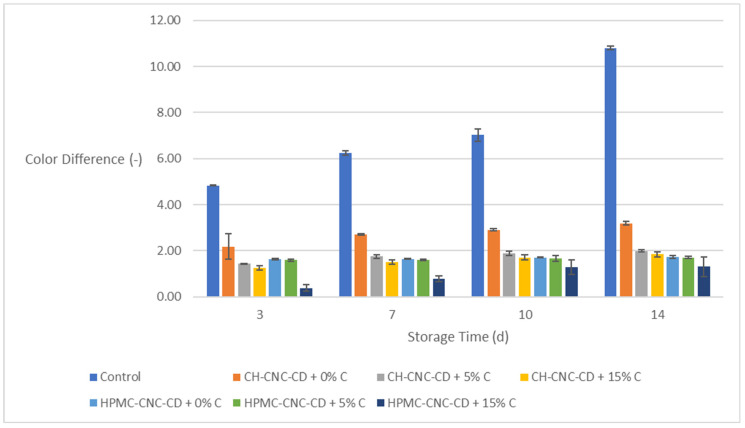
Change in color difference (Δ*E*) of avocados during storage.

As shown in Figure 8, the effect of the edible coatings’ application is significant, since, in all cases, the coated samples (with and without C) showed minimal changes (Δ*Ε* values generally below 3). On the other side, on the 14th day of storage, the control samples reached almost the value of 11. This is a very positive result, as coatings help maintain the attractive appearance/color of the fruits, which play an important role in consumers’ preference.

#### 2.4.3. Hardness of the Avocado Samples

**Figure 9:**
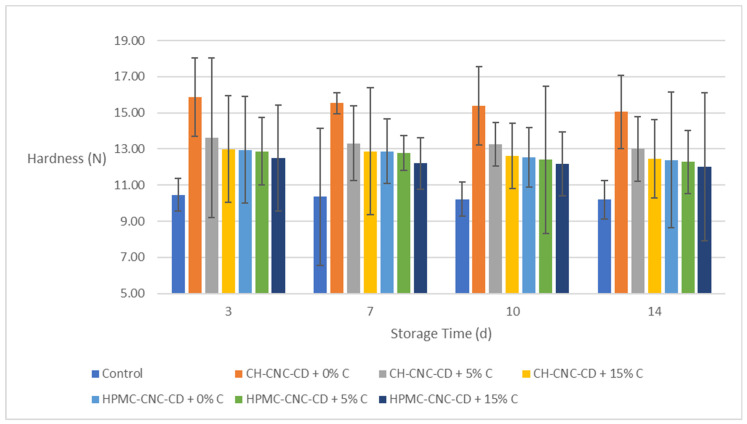
Change in hardness of avocados during storage.

On day 0 (before storage), the hardness of the control sample was measured at 12.164 N. All coated samples (with and without C) retained a significantly higher hardness than the control samples (above 12 N in all cases, even on the 14th day, against 10 N for the control samples on the same day). Additionally, samples coated with CH-CNC-CD exhibited higher hardness values than those with HPMC-CNC-CD, apparently due to the higher mechanical strength of CH (Figure 9).

#### 2.4.4. Peroxide Value (*PV*) of the Avocado Samples

**Figure 10:**
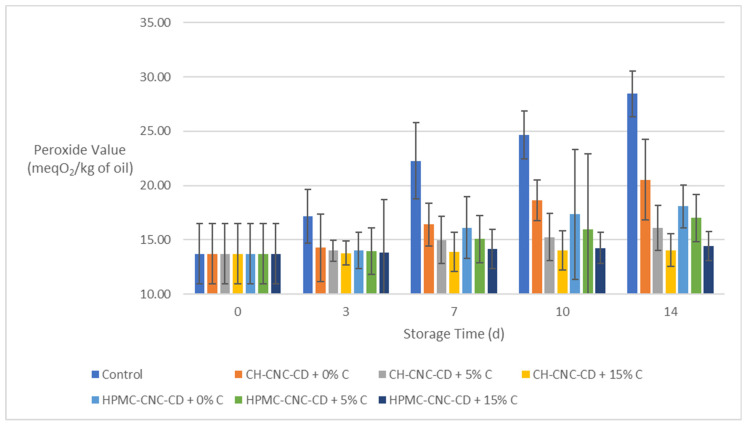
Change in peroxide value (*PV*) of avocados during storage.

During food storage, the peroxide value (*PV*) generally increases due to the oxidation reactions that occur. Figure 10 shows the changes in *PV* during storage for all avocado samples. As expected, the control samples showed the greatest *PV* increase. Also, samples with C containing coatings showed the smallest increases in *PV*, which was noticeable as early as the 3rd day of storage, thus confirming the importance of imparting antioxidant capacity to the films in order to extend shelf life and maintain the quality of fresh-cut fruits. Indeed, the incorporation of 15% *v*/*v* C, retained *PV* at the same levels throughout storage (about 14 meqO_2_/kg of oil) for both types of coatings. This result confirms the antioxidant activity of C that was also observed during the examination of the films themselves (Section 2.2.3). Finally, it can be seen in the diagram that the type of film (CH or HPMC) had no significant effect on this property.

#### 2.4.5. Statistical Analysis of the Results for the Avocado Samples

Table S2 in the Supplementary Materials presents the outcome of the statistical analysis of the results for avocado samples with respect to storage time and the type of sample (control or coated). According to Table S2, all properties changed statistically significantly over storage time in a proportional way in all cases except for Δ*E* between the 7th day and the 10th day of storage. As with strawberries, so in the case of avocados, the coated samples remained satisfactory until the end of storage.

Regarding the influence of the type of sample (control or coated with each of the six different coatings), it emerged that the control samples had statistically significantly different values in Δ*E*, hardness, and *PV* compared with the coated ones. In the case of WL, the control samples did not show statistically significantly different values from the coated ones, expect from those with HPMC-CNC-CD + 0% C and HPMC-CNC-CD + 5% C. In most instances (except for hardness), samples containing the highest C proportion (15% *v*/*v*) exhibited better values compared to the rest of the samples for both types of films (CH-CNC-CD and HPMC-CNC-CD). This was particularly evident for *PV*, which was the property of the highest interest in this case.

## 3. Materials and Methods

### 3.1. Materials

For the preparation of the NE, the following materials were used: olive pomace oil (OPO) from Minerva S.A. (Athens, Greece) and polyoxyethylene (20) sorbitan monopalmitate [Tween 40 (Τ40), PubChem: 87560417] from Acros Organics (Geel, Belgium). 

For the preparation of the composite edible films, the following materials were used: high-molecular-weight CH (deacetylated chitin, ≥75% deacetylated, molecular weight 310,000–375,000 Da, Poly[D-glucosamine]) from Sigma-Aldrich (St. Louis, MO, USA), acetic acid (glacial 99–100% a.r.) from Chem-Lab NV (Zedelgem, Belgium), HPMC (methoxyl 28–30% and hydroxypropyl 7–12%) from Alfa Aesar (Ward Hill, MA, USA), CNC from CelluForce (Montreal, QC, Canada), CD from Acros Organics (Geel, Belgium) and C (98.5% pudicitia, molecular formula: C_8_H_10_N_4_O_2_, PubChem CID: 2519, molecular weight: 194.19 g/mol) from Acros Organics (Geel, Belgium). 

CNC specifications: molecular formula: [(C_6_O_5_H_10_)_22-28_SO_3_Na]_4-6_, specific surface area: 400 m^2^/g, molecular weight (g/mol): 14,700–27,850, particle size: 1–50 μm, particle diameter (crystallite): 2.3–4.5 nm (by atomic force microscopy, AFM), particle length (crystallite): 44–108 nm (by AFM), crystalline fraction: 0.88 (by X-ray diffraction, XRD) and crystallite density: 1.5 g/cm^3^. 

CD specifications: molecular formula: C_42_H_70_O_35_, molecular weight (g/mol): 1134.99, solubility in water: soluble, specific rotation condition: +150.00 (25 °C c = 1.5, in water) and purity: 98%. 

The strawberries (Victory variety) were obtained from the company Ktima Psoni (Nea Manolada, Greece) and the avocados (Bacon variety) from the company Minarakis (Chania, Greece).

### 3.2. Preparation of Functional Composite Edible Films 

Initially, NE and C solution were prepared in liquid form:

- The NE was prepared with 10% *w*/*w* OPO, 6% Tween 40 and 84% H_2_O. To prepare the liquid phase, the emulsifier (Tween 40) was mixed with H_2_O and was stirred with a magnetic stirrer for 1 h. Then, the lipid phase (OPO) was added, and the final mixture was homogenized for 10 min at a speed of 10,000 rpm, using a high-speed homogenizer (CAT Unidrive 1000, CAT Scientific, Paso Robles, CA, USA). Finally, the emulsion was sonicated for 10 min at 45% amplitude, using a 20 kHz high intensity ultrasonic processor (VC 400, Sonic & Materials, Newtown, CT, USA).
- The C solution was prepared by dissolving C powder in deionized water at 1% *w*/*v*.

Subsequently, solutions of CH-CNC-CD and HPMC-CNC-CD (both in proportions of 50%-37.5%-12.5%) were prepared as described in Bizymis et al. [40,41]. Each of the above two liquid components (NE, C) was then added individually to separate amounts of the CH-CNC-CD and HPMC-CNC-CD solutions, at ratios of 5, 10 and 15% *v*/*v*. Following this, the resulting solutions were homogenized and degassed, and 20 mL aliquots were placed in Petri dishes, where they were allowed to dry as described in Bizymis et al. [8]. Thereafter, the films were maintained at room temperature and humidity. 

### 3.3. Measurements in the Composite Edible Films

#### 3.3.1. Mechanical Properties

The evaluation of the mechanical properties was based on the ASTM D882-10 [80] standard. The Texture Analysis was performed with a TA-XT2i Texture Analyzer (Stable Micro Systems, Godalming, UK). A cylindrical probe with a diameter of 5 mm was chosen in order to obtain targeted measurements on the films without causing extensive damage to their surface. The procedure followed for the measurements was as described in Bizymis et al. [40].

#### 3.3.2. Color

The color of the films was determined with the help of a CR-200 Colorimeter (Konica Minolta, Sutton-in-Ashfield, UK). For this purpose, the color parameters *L* (Luminosity), *a* (Red–Green) and *b* (Yellow–Blue) of the Cielab scale were measured. A white calibration plate was used as a standard (*L*_0_, *a*_0_ and *b*_0_). 

The chrome (C*) and the color difference (Δ*Ε*) of the films were derived from the following equations [66,81]:

(1) $$C^{*}=\sqrt{a^{2}+b^{2}}$$

(2) $$\Delta E=\sqrt{{\left(\Delta L\right)}^{2}+{\left(\Delta a\right)}^{2}+{\left(\Delta b\right)}^{2}}$$

where Δ*L* = *L* − *L*_0_, Δ*a* = *a* − *a*_0_ and Δ*b* = *b* − *b*_0_.

#### 3.3.3. Oxygen Permeability 

The oxygen permeability was measured in a liquid analysis system, based on the ASTM D3985-05 [82] standard. The procedure was performed as described in Bizymis et al. [40] and Vogel [83]. Briefly, after passing through each film, oxygen was transferred to the liquid analysis system with the help of nitrogen. For this purpose, two metal cups were used to seal both sides of the film in order to supply oxygen and nitrogen, respectively. In the liquid analysis system, iodometry was applied and the volume of the standard thiosulphate solution that was consumed during titration corresponded to the amount of the O_2_ that had passed through the film (1 mL of the solution corresponded to 1 mg of O_2_).

The entire procedure and the relevant equations are described analytically in Bizymis et al. [40].

The oxygen permeability (*OP*) of the films was derived from the following equation:

(3) $$OP=\frac{m\cdot d\;}{A\cdot t\cdot \Delta P}$$

where *m* was the amount of O_2_ that passed through the film over the time interval *t*, *d* was the thickness of the film, *A* was the area of the film*,* and Δ*P* was the difference of the pressure of O_2_ between each side of the film.

#### 3.3.4. Water Vapor Permeability

The measurement of water vapor permeability was based on the method of Bertuzzi et al. [84] and Bizymis et al. [40]. Briefly, glass cups filled with distilled water and sealed with the edible films and were put into a desiccator containing saturated sodium chloride (NaCl) solution of 75% relative humidity. Movement of water vapor consequently occurred, tending to bring the humidity of the two film sides to the same level. The temperature in the desiccator was 40 °C and no ventilation was used. For 24 h, the weight changes of the cups were periodically measured. 

The Δ*m*/Δ*t* ratio was calculated, using linear regression.

The water vapor permeability (*WVP*) of the films was derived from the following equation:

(4) $$WVP=\frac{\Delta m\;}{\Delta t\;}\cdot \frac{l\;}{A\cdot \Delta P\;}$$

where Δ*m* was the amount of water vapor which passed through the film over the time interval Δ*t*, *l* was the thickness of the film, *A* was the area of the film, and Δ*P* was the difference of the vapor pressure across the film.

#### 3.3.5. Antioxidant Activity in Functional Composite Edible Films with Incorporation of C

To measure the antioxidant activity of the functional films, the 2,2-Diphenyl-1-picrylhydrazyl (*DPPH*) assay was used, as described below: 

Initially, 25 mg of the film was dissolved in 3 mL of deionized water. Thereafter, 1 mL of the resulting solution was mixed with 1 mL of *DPPH* solution and stirred using a Vortex device. The final sample was left in the dark for 30 min. The absorbance (*Abs*) of the samples at the wavelength of 515 nm was then recorded, as well as the absorbance (*Abs*) of the *DPPH* solution at the same wavelength. 

The antioxidant activity (*AA*) was derived from the following equation:

(5) $$AA=\frac{Abs_{DPPH}-Abs_{sample}}{Abs_{DPPH}}\cdot 100\;\;\;$$

### 3.4. Preparation of Strawberry Samples 

Strawberry samples were prepared for coating by removing their leaves, washing and drying. In parallel, CH-CNC-CD and HPMC-CNC-CD solutions (both in the ratio of 50%-37.5%-12.5%) were prepared with the incorporation of NE in amounts of 0, 5 and 15% *v*/*v*, as described in Section 3.2. The strawberry samples were immersed in the solutions for 3 min and thereafter they were allowed to dry at room temperature for 30 min. All samples were then packaged into PET/AL/PE (polyethylene terephthalate/aluminum foil/polyethylene) bags (4 per bag), in an atmospheric air environment (82% nitrogen and 18% oxygen). The bags were sealed and preserved under cold storage (0–3 °C). These conditions were chosen as the most suitable for the type and duration of the experiments. Blank control samples without coating were also stored under the same conditions. 

### 3.5. Preparation of Avocado Samples 

Avocado samples were previously washed and dried, their core and peel were removed, and they were cut into 1 cm × 1 cm × 1 cm cubes. In parallel, CH-CNC-CD and HPMC-CNC-CD solutions (both in the ratio of 50%-37.5%-12.5%) were prepared with C incorporation in amounts of 0, 5 and 15% *v*/*v*, as described in Section 3.2. The avocado samples were immersed in the solutions for 3 min, and thereafter, they were allowed to dry at room temperature for 30 min. All samples were then packaged into PET/AL/PE (polyethylene terephthalate/aluminum foil/polyethylene) bags (10 cubes per bag), in an atmospheric air environment (82% nitrogen and 18% oxygen). The bags were sealed and stored in cold storage (0–3 °C). These conditions were chosen for the same reasons as in the case of strawberries. Blank control samples without coating were also stored under the same conditions. 

### 3.6. Measurements in Strawberry and Avocado Samples

#### 3.6.1. Weight Loss

The samples were weighed immediately after treatment (storage time 0 day) and at different subsequent dates over a two-week period.

The weight loss (*WL*) was derived from the following equation:

(6) $$WL=\frac{m_{0}-m_{t}}{m_{0}}\cdot 100\;$$

where *m*_0_ is the result of the weight measurement of the samples at storage time 0 day and *m_t_* is the result of the weight measurement of the same samples at the sampling date (3rd, 7th, 10th and 14th day).

#### 3.6.2. Color Difference

The color difference (Δ*E*) of the samples, at different dates over the two-week period, was calculated as in Section 3.3.2. except that *L*_0_, *a*_0_ and *b*_0_ were the color parameters of the control samples at storage time 0 day.

#### 3.6.3. Hardness

The hardness of the samples was measured at different dates over the two-week period with the use of a TA-XT2i Texture Analyzer (Stable Micro Systems, UK), which had a suitable cutting blade attached. The penetration depth was selected to be 5 mm, with a crosshead speed of 1 mm/s. The curves of force (N) versus deformation (mm) were derived from the Texture Expert Exceed 2.6 software and the maximum force (N) defined the hardness of the samples. 

#### 3.6.4. Quantity of CO_2_ and O_2_ in the Packages of the Strawberry Samples

To calculate the changes in O_2_ permeability, the quantity of CO_2_ and O_2_ inside the packages (PET/AL/PE bags) of the strawberry samples was measured, at different intervals over the two-week period, using the gas composition measurement device PBI Dansensor (Antylia Scientific, Vernon Hills, IL, USA).

#### 3.6.5. Moisture of Strawberry Samples

To determine the moisture content of the strawberry samples, their weight loss was measured at different time points over the two-week period. Specifically, every time a sliced strawberry was placed in a pre-weighed weighing vial and was weighed. Subsequently, the weighing vial with the sliced strawberry was left in a drying oven at 110 °C for 24 h. Thereafter, it was removed from the oven and re-weighed. 

Moisture (*M_w_*) was derived from the following equation:

(7) $$M_{w}=\frac{m_{0}-m}{m_{0}}\cdot 100\;\;$$

where *m*_0_ is the initial weight of the strawberry (initial gross weight − weighing vial weight) and *m* is the final weight of the strawberry (final dry gross weight − weighing vial weight).

#### 3.6.6. Degree of Oxidation in Avocado Samples

The calculation of the degree of oxidation of the avocado oil was carried out in three-day cycles, at different time points over the two-week period, by determining the number of peroxides (peroxide value, *PV*), which expressed the content of peroxidically combined oxygen and consequently the degree of autoxidation. The method was iodometric, in which the hydroperoxides reacted with hydrogen iodine and the resulting iodine was titrated with a 0.01 N Na_2_S_2_O_3_ solution. Specifically, in every cycle, on the first day, the avocado sample was placed in a drying chamber at a temperature of 60 °C, to remove excess moisture. On the second day, the sample was mixed with hexane at a ratio of 1/10 *v*/*v* and was stirred for 1 h. Then, the liquid part was collected and was left in a drain for 24 h, so that the hexane would evaporate and only the extract (oil) would remain. On the third day, the extract (oil) was pipetted into a conical flask and weighed on an analytical balance, and then, 30 mL of a 3:2 acetic acid–chloroform mixture and 0.5 mL of saturated KI were added. The conical flask was shaken for 1 min and placed in a shaded place for 5 min, and then, 30 mL of H_2_O and 0.5 mL of starch indicator were added. The formed I_2_ was titrated with a 0.01 N Na_2_S_2_O_3_ solution. The titration was completed with the disappearance of the blue color in the titrated solution. The titration was also carried out for the corresponding blank solution (same reagents but not containing oil).

The number of peroxides was derived from the equation:

(8) $$PV=\frac{\left(S-B\right)\cdot N\cdot 1000}{m}\;\;$$

where *S* and *B* are the volume (mL) of the Na_2_S_2_O_3_ solution consumed in the case of the sample and the blank solution, respectively, *N* is the normality of the Na_2_S_2_O_3_ solution (0.01 N), and *m* is the weight of the oil sample.

### 3.7. Statistical Analysis

Statistical data processing was performed by using the Statistica 13.0 software (StatSoft, Inc., Tulsa, OK, USA). In order to evaluate each experimental factor, analysis of variance (ANOVA) was performed. Significant differences were considered at the *p* < 0.05 level. A Duncan’s multiple range test indicated the cases of significantly different levels between each set of means.

## 4. Conclusions

This study demonstrates that functional CH-CNC-CD and HPMC-CNC-CD edible films and coatings enhanced with nanoemulsion (NE) or caffeine (C) exhibit significantly improved properties. The incorporation of NE into edible films markedly enhanced their barrier properties by reducing oxygen permeability (*OP*) and water vapor permeability (*WVP*), while also decreasing color intensity and increasing flexibility. However, NE addition resulted in reduced mechanical strength.

Conversely, the addition of C to the films significantly boosted their antioxidant activity, reduced color intensity, and improved barrier properties by lowering *OP* and *WVP*. In CH-CNC-CD films, C did not significantly affect mechanical properties, whereas in HPMC-CNC-CD films, a slight improvement was observed.

When applied to strawberries, NE-incorporated edible coatings effectively slowed down the respiration process, reducing the consumption of O_2_ and release of CO_2_ within the packaging. NE-coated strawberries experienced lower moisture losses, weight loss (*WL*), and color difference (Δ*E*) compared to control samples. Despite a reduction in firmness, NE-coated strawberries remained firmer than control samples. These findings highlight the beneficial application of NE-containing coatings in reducing O_2_ and H_2_O permeability while maintaining other essential properties.

For avocados, the incorporation of C in edible coatings provided enhanced protection against oxidation. Specifically, coatings with 15% *v*/*v* C maintained a stable peroxide value (*PV*) throughout the storage period. Additionally, both C-containing and non-C coatings helped preserve the color and hardness of the samples, with C significantly improving *WL*, particularly at the 15% *v*/*v* level. Overall, C-enhanced coatings effectively increased antioxidant activity in avocados while maintaining other properties at acceptable to high levels, making their application advantageous.

In summary, the investigated edible films and coatings, particularly those incorporating bioactive components such as NE and C, offer significant potential in food technology. These films contribute to the development of innovative, efficient, cost-effective, and environmentally friendly preservation methods. Therefore, it is highly recommended to expand research in this area, focusing on alternative material combinations, additional properties, and varied application conditions across different types of fresh food.

## Figures and Tables

**Table 1:** Mechanical and optical properties of the composite edible films with incorporation of NE.

Sample	NE (% *v*/*v*)	F (N)	ε (%)	Δ*E* (-)	C* (-)
CH-CNC-CD	0	19.31 ± 1.13 ^aA^	16.78 ± 0.39 ^aA^	47.59 ± 2.86 ^aA^	1.41 ± 0.69 ^aA^
	5	13.51 ± 0.69 ^aB^	23.95 ± 3.54 ^aB^	28.99 ± 0.03 ^aB^	1.15 ± 0.02 ^aA^
	10	10.07 ± 0.54 ^aB^	23.43 ± 0.95 ^aB^	35.39 ± 0.04 ^aB^	0.84 ± 0.03 ^aA^
	15	7.12 ± 0.70 ^aB^	30.03 ± 6.97 ^aB^	29.37 ± 0.20 ^aB^	0.36 ± 0.04 ^aA^
HPMC-CNC-CD	0	13.44 ± 2.39 ^bA^	14.08 ± 1.38 ^bA^	30.37 ± 0.19 ^aA^	1.41 ± 0.15 ^aA^
	5	5.53 ± 0.74 ^bB^	19.48 ± 1.80 ^bB^	34.49 ± 0.14 ^aB^	0.95 ± 0.03 ^aA^
	10	4.80 ± 0.67 ^bB^	23.50 ± 2.05 ^bB^	34.95 ± 0.10 ^aB^	0.58 ± 0.04 ^aA^
	15	3.31 ± 0.67 ^bB^	25.95 ± 2.62 ^bB^	35.10 ± 0.16 ^aB^	0.20 ± 0.02 ^aA^

Values are presented as mean ± standard deviation. Different letters in the same column indicate significant differences (*p* < 0.05) according to the Duncan’s test difference criterion. Small letters indicate differences in relation to the basic material (CH or HPMC). Capital letters indicate differences in relation to the NE quantity.

**Table 2:** Barrier properties of the composite edible films with incorporation of NE.

Sample	NE (% *v*/*v*)	*OP* (g·s^−1^·Pa^−1^·m^−1^)·10^−12^	*WVP* (g·s^−1^·Pa^−1^·m^−1^)·10^−9^
CH-CNC-CD	0	1.89 ± 0.09 ^aA^	2.26 ± 0.08 ^aA^
	5	1.74 ± 0.01 ^aB^	1.93 ± 0.00 ^aB^
	10	1.40 ± 0.00 ^aC^	1.62 ± 0.00 ^aC^
	15	1.02 ± 0.01 ^aD^	1.22 ± 0.00 ^aD^
HPMC-CNC-CD	0	1.60 ± 0.03 ^bA^	1.72 ± 0.00 ^bA^
	5	1.42 ± 0.03 ^bB^	1.59 ± 0.00 ^bB^
	10	1.24 ± 0.03 ^bC^	1.40 ± 0.00 ^bC^
	15	1.04 ± 0.03 ^bD^	1.13 ± 0.00 ^bD^

Values are presented as mean ± standard deviation. Different letters in the same column indicate significant differences (*p* < 0.05) according to the Duncan’s test difference criterion. Small letters indicate differences in relation to the basic material (CH or HPMC). Capital letters indicate differences in relation to the NE quantity.

**Table 3:** Mechanical and optical properties of the composite edible films with incorporation of C.

Sample	C (% *v*/*v*)	F (N)	ε (%)	Δ*E* (-)	C* (-)
CH-CNC-CD	0	19.31 ± 1.13 ^aA^	16.78 ± 0.39 ^aA^	47.59 ± 2.86 ^aA^	1.41 ± 0.69 ^aA^
	5	19.15 ± 2.80 ^aA^	16.88 ± 2.44 ^aA^	32.68 ± 0.02 ^aB^	0.90 ± 0.03 ^aA^
	10	19.15 ± 1.12 ^aA^	16.90 ± 0.35 ^aA^	33.59 ± 0.04 ^aB^	0.66 ± 0.04 ^aA^
	15	19.27 ± 0.65 ^aA^	16.18 ± 3.08 ^aA^	32.28 ± 0.06 ^aB^	0.63 ± 0.01 ^aA^
HPMC-CNC-CD	0	13.44 ± 2.39 ^bA^	14.08 ± 1.38 ^bA^	30.37 ± 0.19 ^aA^	1.41 ± 0.15 ^aA^
	5	19.02 ± 4.02 ^bA^	16.55 ± 2.05 ^bA^	32.43 ± 0.20 ^aB^	0.92 ± 0.02 ^aA^
	10	19.01 ± 5.92 ^bA^	16.03 ± 2.79 ^bA^	32.31 ± 0.23 ^aB^	0.87 ± 0.06 ^aA^
	15	18.67 ± 3.37 ^bA^	16.28 ± 1.73 ^bA^	31.27 ± 0.03 ^aB^	0.62 ± 0.15 ^aA^

Values are presented as mean ± standard deviation. Different letters in the same column indicate significant differences (*p* < 0.05) according to the Duncan’s test difference criterion. Small letters indicate differences in relation to the basic material (CH or HPMC). Capital letters indicate differences in relation to the C quantity.

**Table 4:** Barrier properties of the composite edible films with incorporation of C.

Sample	C (% *v*/*v*)	*OP* (g·s^−1^·Pa^−1^·m^−1^)·10^−12^	*WVP* (g·s^−1^·Pa^−1^·m^−1^)·10^−9^
CH-CNC-CD	0	1.89 ± 0.09 ^aA^	2.26 ± 0.08 ^aA^
	5	1.25 ± 0.01 ^aB^	1.43 ± 0.00 ^aB^
	10	1.22 ± 0.01 ^aB^	1.40 ± 0.00 ^aB^
	15	1.20 ± 0.01 ^aB^	1.38 ± 0.00 ^aB^
HPMC-CNC-CD	0	1.60 ± 0.03 ^bA^	1.72 ± 0.00 ^bA^
	5	1.23 ± 0.01 ^bB^	1.42 ± 0.00 ^bB^
	10	1.19 ± 0.01 ^bB^	1.40 ± 0.00 ^bB^
	15	1.16 ± 0.01 ^bB^	1.36 ± 0.00 ^bB^

Values are presented as mean ± standard deviation. Different letters in the same column indicate significant differences (*p* < 0.05) according to the Duncan’s test difference criterion. Small letters indicate differences in relation to the basic material (CH or HPMC). Capital letters indicate differences in relation to the C quantity.

**Table 5:** Antioxidant activity (*AA*) of composite edible films with incorporation of C.

Sample	C (% *v*/*v*)	Antioxidant Activity (%)
CH-CNC-CD	0	81.04 ± 1.64 ^aA^
	5	84.06 ± 0.66 ^aB^
	10	86.46 ± 0.33 ^aC^
	15	88.54 ± 0.22 ^aD^
HPMC-CNC-CD	0	72.14 ± 0.44 ^bA^
	5	76.93 ± 0.22 ^bB^
	10	79.80 ± 0.33 ^bC^
	15	81.19 ± 0.11 ^bD^

Values are presented as mean ± standard deviation. Different letters in the same column indicate significant differences (*p* < 0.05) according to the Duncan’s test difference criterion. Small letters indicate differences in relation to the basic material (CH or HPMC). Capital letters indicate differences in relation to the C quantity.

## Data Availability

All data generated or analyzed during this study are included in this published article.

## Supplementary Materials

The following supporting information can be downloaded at: https://www.mdpi.com/article/10.3390/molecules29163754/s1, Table S1: Results with statistical analysis for coated strawberry samples; Table S2: Results with statistical analysis for coated avocado samples.

## Abbreviations

AA	antioxidant activity
Abs	absorbance
AFM	atomic force microscopy
AL	aluminum foil
C	caffeine
C*	chrome
CD	beta-cyclodextrin
CH	chitosan
CNC	cellulose nanocrystals
DPPH	2,2-Diphenyl-1-picrylhydrazyl
EFSA	European Food Safety Authority
F	maximum breaking force
FDA	Food and Drug Administration
FTIR	Fourier-transform infrared spectroscopy
HPMC	hydroxypropyl methylcellulose
M_w_	moisture
NE	pomace oil-based nanoemulsion
OP	oxygen permeability
OPO	olive pomace oil
PE	polyethylene
PET	polyethylene terephthalate
PV	peroxide value
ROS	reactive oxygen species
SEM	scanning electron microscopy
Τ40	Tween 40
WL	weight loss
WVP	water vapor permeability
XRD	X-ray diffraction
ΔE	color difference
ε	elongation
