# Supplementary material for: Development of Functional Composite Edible Films or Coatings for Fruits Preservation with Addition of Pomace Oil-Based Nanoemulsion for Enhanced Barrier Properties and Caffeine for Enhanced Antioxidant Activity
Source: Molecules. 2024 Aug 8;29(16):3754. doi: 10.3390/molecules29163754 (PMC11356815; doi:10.3390/molecules29163754)
Supplement: Supplementary file 1 [file molecules-29-03754-s001.zip › molecules-3105644-supplementary.pdf]

Supplementary Materials

Table S1: *Results with statistical analysis for coated strawberry samples.*

Storage Time (days)	Sample	WL (%)	ΔE (-)	Hardness (N)	Quantity of O_2 (%)	Quantity of CO_2 (%)	M_w (%)
3	Control	0.51 ± 0.01 aA	7.04 ± 0.03 aA	1.545 ± 0.064 aA	10.300 ± 0.000 aA	1.000 ± 0.000 aA	90.7587 ± 0.3172 aA
	CH-CNC-CD + 0% NE	0.45 ± 0.01 bA	4.62 ± 0.22 bA	4.294 ± 0.402 bA	14.000 ± 0.273 bA	0.000 ± 0.000 bA	92.3378 ± 0.1192 bA
	CH-CNC-CD + 5% NE	0.39 ± 0.01 cA	3.15 ± 0.00 cA	2.511 ± 0.246 cA	14.230 ± 0.057 bA	0.000 ± 0.424 cA	92.4754 ± 0.0861 bA
	CH-CNC-CD + 15% NE	0.34 ± 0.01 cA	1.85 ± 0.03 dA	2.507 ± 0.295 cA	15.395 ± 0.078 cA	0.000 ± 0.424 cA	92.6470 ± 0.0196 cA
	HPMC-CNC-CD + 0% NE	0.42 ± 0.01 cA	4.17 ± 0.07 eA	3.735 ± 0.088 dA	12.885 ± 0.064 dA	0.000 ± 0.354 bA	92.2525 ± 0.1615 dA
	HPMC-CNC-CD + 5% NE	0.38 ± 0.01 cA	3.36 ± 0.16 cA	2.791 ± 0.071 cA	13.430 ± 0.057 eA	0.000 ± 0.424 cA	92.5098 ± 0.0389 bA
	HPMC-CNC-CD + 15% NE	0.33 ± 0.01 dA	2.21 ± 0.02 dA	2.630 ± 0.757 cA	14.945 ± 0.078 fA	0.000 ± 0.354 dA	92.7318 ± 0.0343 cA
7	Control	0.85 ± 0.01 aB	7.33 ± 0.07 aB	1.525 ± 0.039 aB	5.955 ± 0.428 aB	9.400 ± 0.808 aB	88.5041 ± 0.1862 aB
	CH-CNC-CD + 0% NE	0.61 ± 0.01 bB	4.89 ± 0.03 bB	4.008 ± 0.345 bB	11.039 ± 0.055 bB	7.900 ± 0.919 bB	91.6621 ± 0.2099 bB
	CH-CNC-CD + 5% NE	0.52 ± 0.01 cB	3.47 ± 0.07 cB	2.388 ± 0.165 cB	12.037 ± 0.052 bB	6.760 ± 0.919 cB	92.1375 ± 0.6205 bB
	CH-CNC-CD + 15% NE	0.47 ± 0.01 cB	2.57 ± 0.14 dB	2.340 ± 0.470 cB	13.044 ± 0.062 cB	5.500 ± 0.778 cB	92.4369 ± 0.1529 cB
	HPMC-CNC-CD + 0% NE	0.53 ± 0.01 cB	4.64 ± 0.04 eB	3.457 ± 0.359 dB	10.022 ± 0.030 dB	8.950 ± 0.636 bB	91.4916 ± 0.1252 dB
	HPMC-CNC-CD + 5% NE	0.49 ± 0.01 cB	3.65 ± 0.08 cB	2.509 ± 0.554 cB	11.028 ± 0.040 eB	7.300 ± 0.849 cB	91.9511 ± 1.2347 bB
	HPMC-CNC-CD + 15% NE	0.40 ± 0.01 dB	2.57 ± 0.07 dB	2.200 ± 0.260 cB	12.850 ± 0.071 fB	4.050 ± 0.354 dB	92.4754 ± 0.2458 cB
10	Control	1.11 ± 0.01 aC	7.98 ± 0.09 aC	1.482 ± 0.088 aC	0.045 ± 0.064 aC	28.350 ± 0.919 aC	86.6608 ± 0.1561 aC
	CH-CNC-CD + 0% NE	0.78 ± 0.01 bC	5.19 ± 0.17 bC	3.840 ± 0.450 bC	7.569 ± 0.054 bC	23.200 ± 0.990 bC	90.9940 ± 0.4227 bC
	CH-CNC-CD + 5% NE	0.67 ± 0.01 cC	4.46 ± 0.20 cC	2.249 ± 0.226 cC	8.029 ± 0.040 bC	20.700 ± 0.849 cC	91.7122 ± 0.0572 bC
	CH-CNC-CD + 15% NE	0.62 ± 0.01 cC	2.69 ± 0.07 dC	2.173 ± 0.117 cC	9.041 ± 0.058 cC	19.000 ± 0.849 cC	92.2447 ± 0.2191 cC
	HPMC-CNC-CD + 0% NE	0.66 ± 0.01 cC	4.72 ± 0.24 eC	3.291 ± 0.185 dC	6.033 ± 0.046 dC	20.650 ± 0.919 bC	90.6103 ± 0.1400 dC
	HPMC-CNC-CD + 5% NE	0.58 ± 0.01 cC	4.33 ± 0.12 cC	2.156 ± 0.748 cC	7.032 ± 0.045 eC	19.650 ± 0.778 cC	91.4379 ± 0.0765 bC
	HPMC-CNC-CD + 15% NE	0.51 ± 0.01 dC	2.98 ± 0.11 dC	2.066 ± 0.202 cC	8.223 ± 0.081 fC	17.100 ± 0.990 dC	91.9526 ± 0.1086 cC
14	Control	1.58 ± 0.01 aD	8.00 ± 0.09 aC	1.231 ± 0.432 aC	0.037 ± 0.052 aD	30.000 ± 0.849 aD	86.0703 ± 0.1631 aC
	CH-CNC-CD + 0% NE	0.97 ± 0.01 bD	5.35 ± 0.03 bC	3.064 ± 0.045 bC	0.527 ± 0.038 bD	28.050 ± 0.919 bD	90.3730 ± 0.2032 bC
	CH-CNC-CD + 5% NE	0.83 ± 0.01 cD	4.56 ± 0.12 cC	1.746 ± 0.505 cC	0.923 ± 0.032 bD	27.000 ± 1.273 cD	91.1923 ± 0.5535 bC
	CH-CNC-CD	0.74 ± 0.01 cD	3.27 ± 0.20 dC	1.908 ± 0.353 cC	2.024 ± 0.033 cD	26.200 ± 0.849 cD	91.8354 ± 0.3145 cC

+ 15% NE						
HPMC-CNC-CD + 0% NE	0.74 ± 0.01 ^{cD}	4.81 ± 0.10 ^{eC}	2.919 ± 0.346 ^{dC}	0.220 ± 0.028 ^{dD}	27.050 ± 0.778 ^{bD}	90.0514 ± 0.2105 ^{dC}
HPMC-CNC-CD + 5% NE	0.65 ± 0.01 ^{cD}	4.51 ± 0.15 ^{cC}	1.686 ± 0.334 ^{cC}	0.523 ± 0.033 ^{eD}	26.300 ± 0.990 ^{cD}	90.7062 ± 0.2730 ^{bC}
HPMC-CNC-CD + 15% NE	0.56 ± 0.01 ^{dD}	3.18 ± 0.13 ^{dC}	1.579 ± 0.074 ^{cC}	1.018 ± 0.025 ^{fD}	24.500 ± 0.707 ^{dD}	91.2768 ± 0.0056 ^{cC}

[Values are presented as mean ± standard deviation. Different letters in the same column indicate significant differences ($p < 0.05$) according to the Duncan's test difference criterion. Small letters indicate differences in relation to the type of the strawberry samples (control or coated). Capital letters indicate differences in relation to the storage time.]

Table S2: *Results with statistical analysis for coated avocado samples.*

Storage Time (days)	Sample	WL (%)	ΔE (-)	Hardness (N)	PV (meqO_2/kg of oil)
3	Control	0.41 ± 0.01 ^{aA}	4.83 ± 0.01 ^{aA}	10.467 ± 0.916 ^{aA}	17.1507 ± 2.4548 ^{aA}
	CH-CNC-CD + 0% C	0.28 ± 0.01 ^{aA}	2.18 ± 0.56 ^{bA}	15.886 ± 2.168 ^{bA}	14.2567 ± 3.1246 ^{bA}
	CH-CNC-CD + 5% C	0.49 ± 0.01 ^{aA}	1.43 ± 0.02 ^{cA}	13.623 ± 4.404 ^{cA}	13.9978 ± 0.9788 ^{cA}
	CH-CNC-CD + 15% C	0.16 ± 0.01 ^{aA}	1.26 ± 0.11 ^{cA}	12.987 ± 2.945 ^{dA}	13.7587 ± 1.1132 ^{dA}
	HPMC-CNC-CD + 0% C	0.59 ± 0.01 ^{bA}	1.62 ± 0.03 ^{cA}	12.953 ± 2.952 ^{eA}	14.0147 ± 1.6732 ^{cA}
	HPMC-CNC-CD + 5% C	0.57 ± 0.01 ^{bA}	1.59 ± 0.04 ^{cA}	12.874 ± 1.869 ^{fA}	13.9663 ± 2.1298 ^{cA}
	HPMC-CNC-CD + 15% C	0.39 ± 0.01 ^{aA}	0.38 ± 0.13 ^{dA}	12.498 ± 2.930 ^{gA}	13.8266 ± 4.8388 ^{dA}
7	Control	0.55 ± 0.01 ^{aB}	6.25 ± 0.09 ^{aB}	10.349 ± 3.789 ^{aB}	22.2741 ± 3.5110 ^{aB}
	CH-CNC-CD + 0% C	0.88 ± 0.01 ^{aB}	2.70 ± 0.03 ^{bB}	15.528 ± 0.598 ^{bB}	16.4035 ± 1.9924 ^{bB}
	CH-CNC-CD + 5% C	0.60 ± 0.01 ^{aB}	1.75 ± 0.09 ^{cB}	13.316 ± 2.050 ^{cB}	14.9732 ± 2.1532 ^{cB}
	CH-CNC-CD + 15% C	0.66 ± 0.01 ^{aB}	1.50 ± 0.09 ^{cB}	12.875 ± 3.523 ^{dB}	13.8900 ± 1.7961 ^{dB}
	HPMC-CNC-CD + 0% C	0.92 ± 0.01 ^{bB}	1.65 ± 0.01 ^{cB}	12.875 ± 1.787 ^{eB}	16.1044 ± 2.8398 ^{cB}
	HPMC-CNC-CD + 5% C	0.89 ± 0.01 ^{bB}	1.61 ± 0.02 ^{cB}	12.770 ± 0.950 ^{fB}	15.0736 ± 2.1690 ^{cB}
	HPMC-CNC-CD + 15% C	0.77 ± 0.01 ^{aB}	0.78 ± 0.12 ^{dB}	12.197 ± 1.439 ^{gB}	14.1375 ± 1.8043 ^{dB}
10	Control	0.70 ± 0.01 ^{aC}	7.02 ± 0.28 ^{aB}	10.219 ± 0.937 ^{aC}	24.6690 ± 2.2083 ^{aC}
	CH-CNC-CD + 0% C	0.88 ± 0.01 ^{aC}	2.90 ± 0.04 ^{bB}	15.385 ± 2.150 ^{bC}	18.6232 ± 1.8501 ^{bC}
	CH-CNC-CD + 5% C	1.13 ± 0.01 ^{aC}	1.90 ± 0.09 ^{cB}	13.259 ± 1.220 ^{cC}	15.2492 ± 2.2028 ^{cC}
	CH-CNC-CD + 15% C	0.91 ± 0.01 ^{aC}	1.70 ± 0.11 ^{cB}	12.625 ± 1.810 ^{dC}	14.0056 ± 1.8006 ^{dC}
	HPMC-CNC-CD + 0% C	1.39 ± 0.01 ^{bC}	1.70 ± 0.01 ^{cB}	12.545 ± 1.642 ^{eC}	17.3409 ± 5.9857 ^{cC}
	HPMC-CNC-CD + 5% C	1.31 ± 0.01 ^{bC}	1.65 ± 0.12 ^{cB}	12.395 ± 4.089 ^{fC}	15.9563 ± 6.9256 ^{cC}
	HPMC-CNC-CD + 15% C	0.87 ± 0.01 ^{aC}	1.28 ± 0.33 ^{dB}	12.189 ± 1.766 ^{gC}	14.2353 ± 1.4188 ^{dC}
14	Control	1.40 ± 0.01 ^{aD}	10.81 ± 0.07 ^{aC}	10.193 ± 1.074 ^{aD}	28.4346 ± 2.1165 ^{aD}
	CH-CNC-CD + 0% C	1.70 ± 0.01 ^{aD}	3.20 ± 0.09 ^{bC}	15.045 ± 2.041 ^{bD}	20.5356 ± 3.6983 ^{bD}
	CH-CNC-CD + 5% C	1.47 ± 0.01 ^{aD}	2.00 ± 0.05 ^{cC}	13.003 ± 1.799 ^{cD}	16.1196 ± 2.0724 ^{cD}
	CH-CNC-CD + 15% C	1.44 ± 0.01 ^{aD}	1.84 ± 0.11 ^{cC}	12.455 ± 2.180 ^{dD}	14.0389 ± 1.5272 ^{dD}
	HPMC-CNC-CD + 0% C	1.83 ± 0.01 ^{bD}	1.74 ± 0.06 ^{cC}	12.380 ± 3.749 ^{eD}	18.0656 ± 1.9653 ^{cD}
	HPMC-CNC-CD + 5% C	1.77 ± 0.01 ^{bD}	1.71 ± 0.04 ^{cC}	12.279 ± 1.741 ^{fD}	16.9963 ± 2.1851 ^{cD}
	HPMC-CNC-CD + 15% C	1.37 ± 0.01 ^{aD}	1.31 ± 0.43 ^{dC}	12.008 ± 4.098 ^{gD}	14.3930 ± 1.3362 ^{dD}

[Values are presented as mean ± standard deviation. Different letters in the same column indicate significant differences ($p < 0.05$) according to the Duncan's test difference criterion. Small letters indicate differences in relation to the type of the avocado samples (control or coated). Capital letters indicate differences in relation to the storage time.]
